# Efficacy and Safety of Minimally Invasive Surgery Versus Open Laparotomy for Interval Debulking Surgery of Advanced Ovarian Cancer After Neoadjuvant Chemotherapy: A Systematic Review and A Meta-Analysis

**DOI:** 10.3389/fonc.2022.900256

**Published:** 2022-07-18

**Authors:** Siyuan Zeng, Yongai Yu, Yuemei Cui, Bing Liu, Xianyu Jin, Zhengyan Li, Lifeng Liu

**Affiliations:** ^1^ Department of Obstetrics and Gynecology, Dalian Municipal Central Hospital, Dalian, China; ^2^ Dalian municipal Central Hospital, China Medical University, Shenyang, China

**Keywords:** Advanced ovarian cancer (AOC), neo-adjuvant chemotherapy (NACT), minimally invasive surgery (MIS), laparotomy, interval debulking surgery (IDS)

## Abstract

**Objective:**

The selection of minimally invasive surgery (MIS) or open laparotomy for ovarian cancer (OC) after neoadjuvant chemotherapy still remains controversial. This study aimed to assess the efficacy and safety of MIS versus open laparotomy following neoadjuvant chemotherapy for advanced OC, so as to provide another option to select optimal surgical procedures for patients with OC.

**Methods:**

Relevant literature studies about the risks of progression or mortality between women receiving MIS and open laparotomy for interval debulking surgery (IDS) were searched in the online databases, including PubMed, Embase, and the Cochrane Library with the following keywords: “ovarian neoplasms”, “minimally invasive surgical procedures”, “laparotomy”, and “neoadjuvant therapy”. Eligible studies were screened out for further meta-analysis.

**Results:**

Six eligible literature studies, with 643 patients in the MIS group and 2,885 patients in the open laparotomy group, were included in this meta-analysis. No significant differences were detected in the overall survival (OS) of patients with OC who were treated with MIS or open laparotomy [hazard ratio (HR) = 0.85; 95% confidence interval (CI) = 0.59–1.23; heterogeneity: P = 0.051, I^2^ = 57.6%]. However, the progression-free survival (PFS) was significantly higher in patients with OC treated with MIS than those treated with laparotomy (HR = 0.73; 95% CI = 0.57 to 0.92; heterogeneity: P = 0.276, I^2^ = 22.4%). The completeness of debulking removal (R0 rate) in the open laparotomy group was not statistically higher compared with the control group (RR = 1.07; 95% CI = 0.93 to 1.23; heterogeneity: P = 0.098, I^2^ = 52.3%), and no significant differences in residual disease of ≤1 cm (R1) (RR = 1.08; 95% CI = 0.91 to 1.28; heterogeneity: P = 0.330, I^2^ = 12.6%) and postoperative complications were found between the two groups (RR = 0.72; 95% CI = 0.34 to 1.54; heterogeneity: P = 0.055, I^2^ = 60.6%). Furthermore, the length of stays in hospital was significantly shorter in patients with OC treated with MIS than those treated with open laparotomy (Standard Mean Difference (SMD) = −1.21; 95% CI = −1.78 to −0.64; heterogeneity: P < 0.001, I^2^ = 92.7%].

**Conclusions:**

For IDS after NACT in patients with advanced OC, complete cytoreductive surgery with MIS is another feasible and effective choice

**Systematic Review Registration:**

https://www.crd.york.ac.uk/prospero/display_record.php?ID=CRD42022298519, identifier CRD42022298519

## Introduction

Ovarian cancer (OC) is one of the most fatal gynecologic cancers in women (1). Epithelial OC is difficult to be detected early, and nearly 75% of patients with OC are detected at advanced stage (2, 3).

Traditionally, in patients with advanced OC, the standard treatment relied on laparotomy-based primary debulking surgery (PDS), followed by adjuvant platinum-based chemotherapy. Another recently emerged and developed treatment strategy is neoadjuvant chemotherapy (NACT) and interval debulking surgery (IDS), particularly in patients with high perioperative risk or limited likelihood of achieving adequate cytoreduction at PDS (4). According to two phase III clinical trials, patients with advanced OC receiving NACT and IDS have equivalent survival to those who received PDS and adjuvant chemotherapy (5, 6), as well as lower incidence of related morbidity and mortality.

However, in patients with advanced OC with large tumor lesions and/or complicated medical conditions, sophisticated surgical treatments may increase the risks of severe postoperative morbidity and mortality (M/M) (7). With the advancement of diagnostic and therapeutic technologies, minimally invasive surgery (MIS) is gradually recognized across gynecological debulking surgery (8). In patients with OC with complete response to NACT, a phase II clinical trial demonstrated that MIS is a feasible, effective, safe, and alternative procedure for IDS (9). According to the most recent OC guidelines (10), MIS is of great importance in debulking surgery of OC. Despite the fact that several studies have assessed the efficacy and safety of MIS and laparotomy in patients with OC after NACT (11–18), the results are still controversial.

To investigate the feasibility of MIS, this study systematically analyzed the comprehensive studies and compared the efficacy and safety of MIS and laparotomy in advanced patients with OC after NACT.

## Methods

### Searching Strategy and Selection Criteria

This meta-analysis was performed by following the Preferred Reporting Items for Systematic Reviews and Meta-analysis (PRISMA) criteria (19). The following keywords were searched in the online databases, such as PubMed, Embase, and Cochrane Library: “neoadjuvant therapy”, “minimally invasive surgery”, “open laparotomy”, and “interval debulking surgery”. Selection criteria are as follows: 1) patients with advanced OC; 2) MIS versus open laparotomy in patients with OC who were treated with IDS after NACT; 3) the overall survival (OS), progression-free survival (PFS), completeness of debulking removal (R0), residual disease of ≤1 cm (R1), postoperative complications, and length of stays in hospital were reported; and 4) English-language published literature studies. Exclusion criteria: 1) abstracts without full text; 2) duplicates; and 3) IDS for advanced OC after non-neoadjuvant chemotherapy.

### Data Extraction and Quality Assessment

Two investigators were independently responsible for data extraction, and any disagreements were solved by a third contributor. The following data were extracted using a previously formulated data extraction table: (1) basic information of the literature: title, first author, publication journal, time, etc.; (2) baseline characteristics of subjects: age, case number, follow-up time, International Federation of Gynecology and Obstetrics (FIGO) stage, etc.; (3) chemotherapy cycles, American Society of Anesthesiologists physical status classification system (ASA) score, Response Evaluation Criteria in Solid Tumors (RECIST) response, study types, and key elements of quality evaluation; and (4) outcomes and the measurement data. To assess the quality of retrospective investigations, the nine-star Newcastle-Ottawa scale (NOS) was used, and those with a minimum of six stars were considered as high-quality (20).

### Statistical Analysis

The directly reported outcomes are rare. Therefore, log-rank p-values or Kaplan–Meier survival curves were used to extract the hazard ratio (HR) and the variance of the text (21); the combined risk ratio (RR) or HR and the corresponding 95% confidence interval (CI) were calculated using Stata 14.0; The Q-test was used to assess heterogeneity. If I^2^ < 50%, the fixed-effect model was used to combine the HR or RR of each study; otherwise, the random-effect model was utilized. By removing each study and calculating, the sensitivity analysis was used to determine the related effects of individual studies on the combined results. Begg’s funnel plots were depicted to assess publication bias. *P* < 0.05 was considered to be statistically significant.

## Results

### Eligible Literature Studies

**Figure 1:**
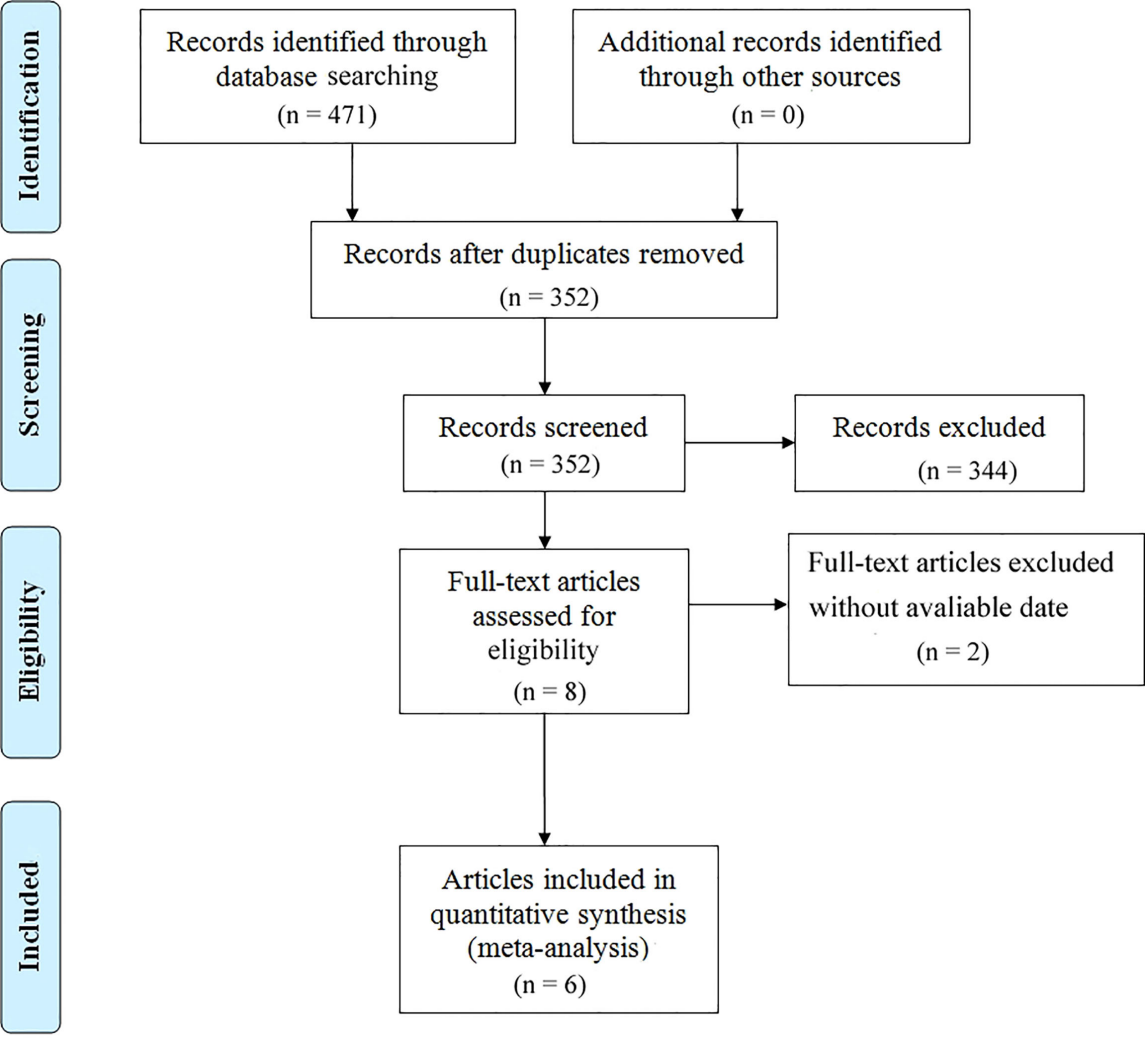
Flow chart of the meta-analysis.

As shown in **Figure 1**, 471 literature studies were initially searched, and 352 remained after excluding duplicates. Through reviewing the titles and abstracts based on the inclusion and exclusion criteria, 344 literature studies were excluded. Finally, six retrospective studies involving 3,528 patients with OC were recruited by reviewing the full text. The basic information of eligible literature studies was listed in **Table 1**. Three studies were performed in America, two in Europe, and one in Asia. The sample size was from 21 to 3,071. The information of chemotherapy cycles was reported in four articles (two reported patients underwent six cycles of NACT and two reported patients underwent less than six cycles of NACT). Two literature studies reported patients’ ASA score. The RECIST response to NACT was investigated in three articles, and there were no significant differences in NACT response between the MIS group and the laparotomy group in the original studies (**Table 1**). Moreover, all the studies were assessed as high-quality on the basis of the NOS (**Supplementary Table 1**).

**Table 1:** Characteristics of studies included in this meta-analysis.

Study, year	Duration	Study design	Surgical procedure	Sample size (T/C)	Age (T/C)	Chemotherapy cycles	ASA score	RECIST response (%)		Stage	Follow-up (Months)	Primary outcome	Secondary outcome	Analysis	NOS
								Complete (T/C)	Partial (T/C)						
Favero, 2015 (12)	2011–2014	Retrospective	Laparoscopy vs. Open	10/11	mean: 58.3 (42–73)/61.3 (41–80)	mean: 6	NR	10 (100)/11 (100)	0 (0)/0 (0)	IIIc–IVa	mean: 20	OS	Postoperative complications, Length of stay	U	7
Alletti, 2016 (16)	2010–2014	Retrospective	Laparoscopy/Robotic vs. Open	30/65	median: 62 (40–81)/59 (48–80)	mean: 6	median: 2 (1-2)	6 (20)/12 (18.5)	24 (80)/53 (81.5)	III–IV	median: 28	PFS	Postoperative complications, Completeness of debulking removal, Residual disease ≤1 cm, Length of stay	M	7
Melamed, 2017 (15)	2010–2012	Retrospective	Laparoscopy vs. Open	450/2621	mean: 63.9 ± 11.7/63.2 ± 11.1	NR	NR	NR	NR	III–IV	median: 32	OS	Completeness of debulking removal, Residual disease ≤1 cm, Length of stay	M	8
Abitbol, 2019	2008–2014	Retrospective	Robotic vs. Open	57/34	median: 65 (24–88)	NR	1 (3.3%); 2 (59.3%); 3(36.3); Unknow (1.1%)	NR	NR	III–IV	median: 37	OS, PFS	Length of stay	U	7
Brown, 2019 (13)	2006–2017	Retrospective	Laparoscopy/Robotic vs. Open	53/104	mean: 66.6 ± 11.0/67.1 ± 9.6	mean: 3.5	NR	9 (17.0)/13 (12.8)	43 (81.1)/81 (79.4)	III–IV	NR	OS, PFS	Postoperative complications, Completeness of debulking removal, Residual disease ≤1 cm, Length of stay	U	8
Zhang, 2021 (11)	2011–2018	Retrospective	Robotic vs. Open	43/50	mean: 66.2/63	mean: 4.2	NR	NR	NR	III–IV	T: median 31.8; C: median 27.0	OS, PFS	Postoperative complications, Completeness of debulking removal, Residual disease ≤1 cm, Length of stay	M	8

ASA, American Society of Anesthesiologists physical status classification system; C, control; M, multivariable; NR, not reported; NOS, Newcastle-Ottawa Scale; OS, overall survival; PFS, progression-free survival; T, test; U, univariable.

### Primary Outcomes

**Figure 2:**
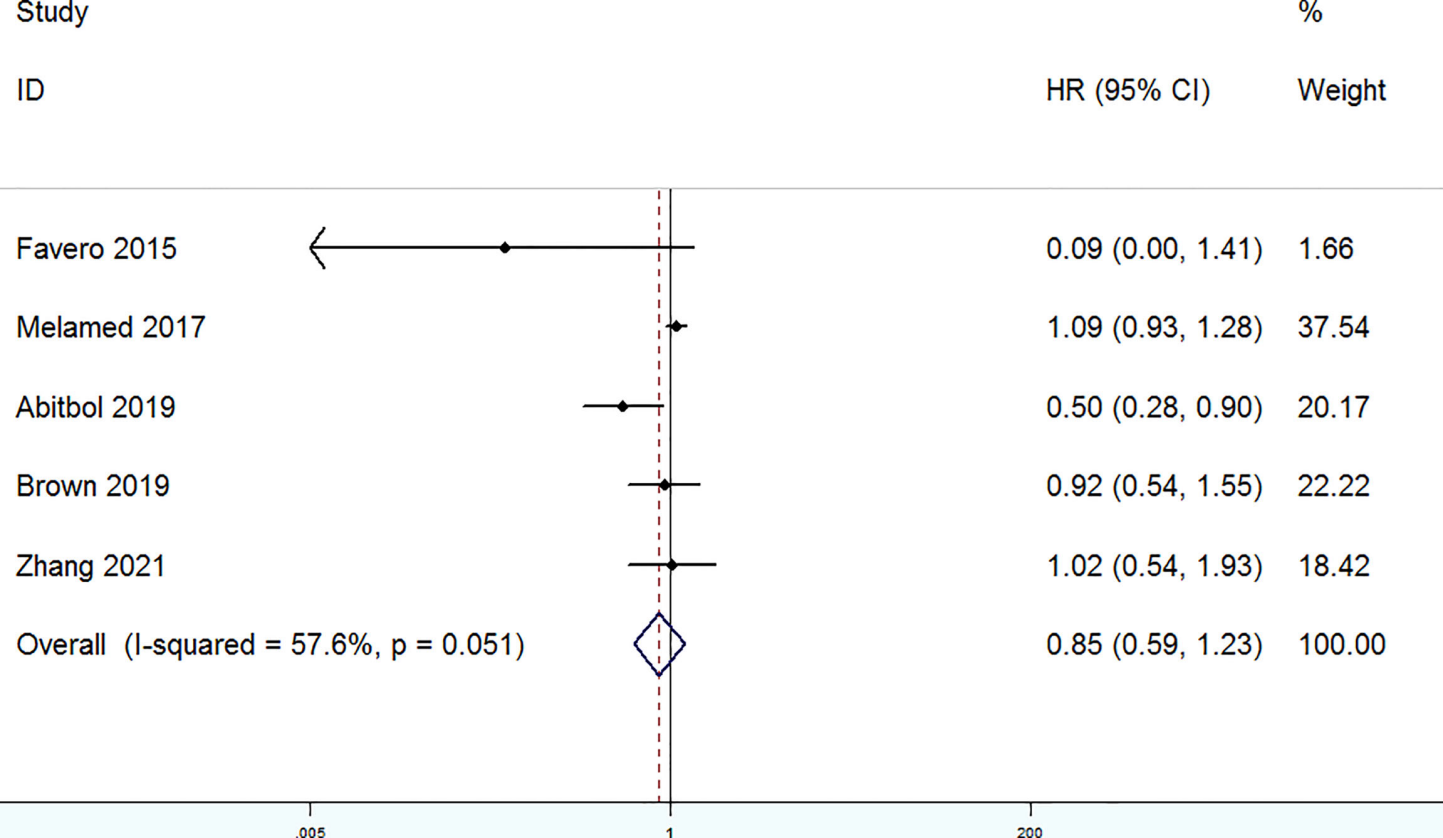
Forest plots for overall survival (OS).

**Figure 3:**
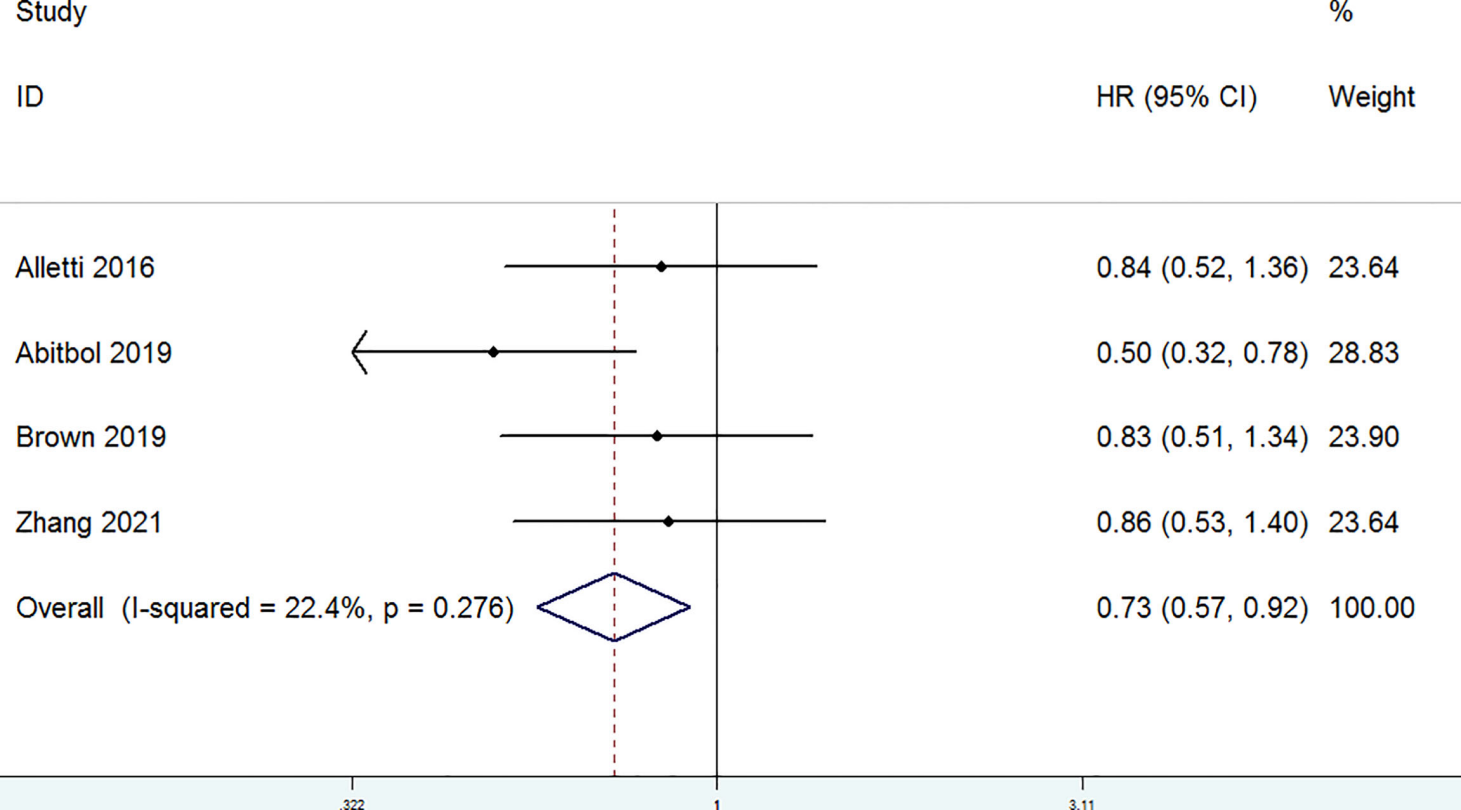
Forest plots for progression-free survival (PFS).

The OS of patients with OC who were treated with MIS versus laparotomy was examined in five studies. It was found that heterogeneity is substantial (*P* = 0.051, I^2^ = 57.6%), so a random-effect model was used. No significant differences in OS were found between patients with OC in the laparotomy group and the MIS group (HR = 0.85, 95% CI = 0.59 to 1.23) (**Figure 2**). In addition, PFS of patients with OC treated with MIS versus open laparotomy was examined in four studies. The fixed-effect model was adopted to examine the pooled findings due to the non-significant heterogeneity (*P* = 0.276, I^2^ = 22.4%). In patients with OC after NACT, the MIS group significantly enhanced PFS, compared with the open laparotomy group (HR = 0.73, 95% CI = 0.57 to 0.92) (**Figure 3**).

### Secondary Outcomes

**Figure 4:**
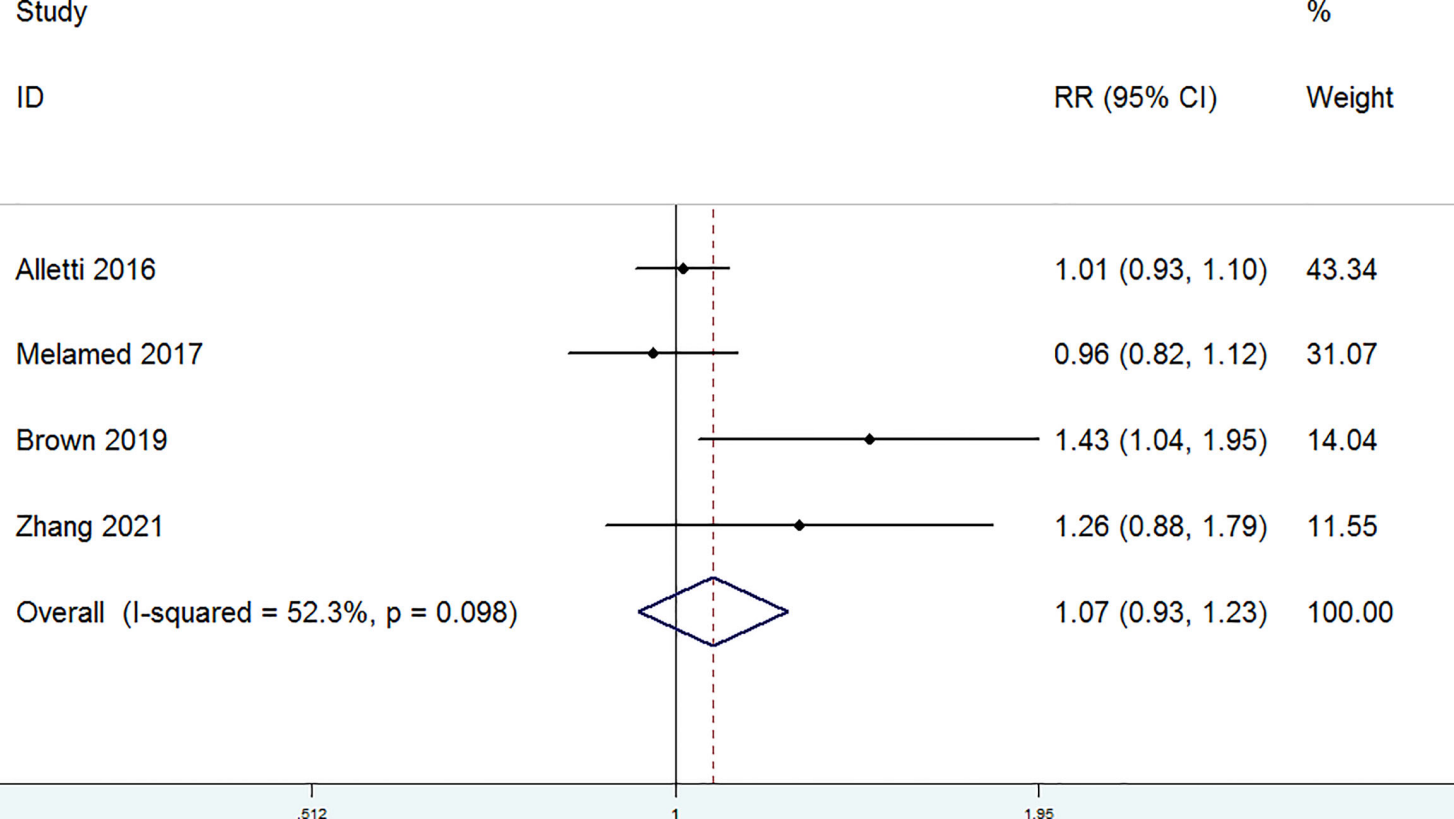
Forest plots for completeness of debulking removal (R0).

**Figure 5:**
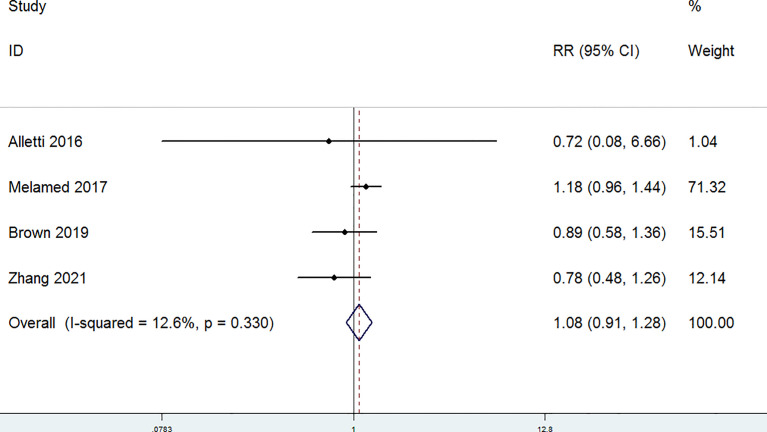
Forest plots for residual disease ≤1 cm (R1).

**Figure 6:**
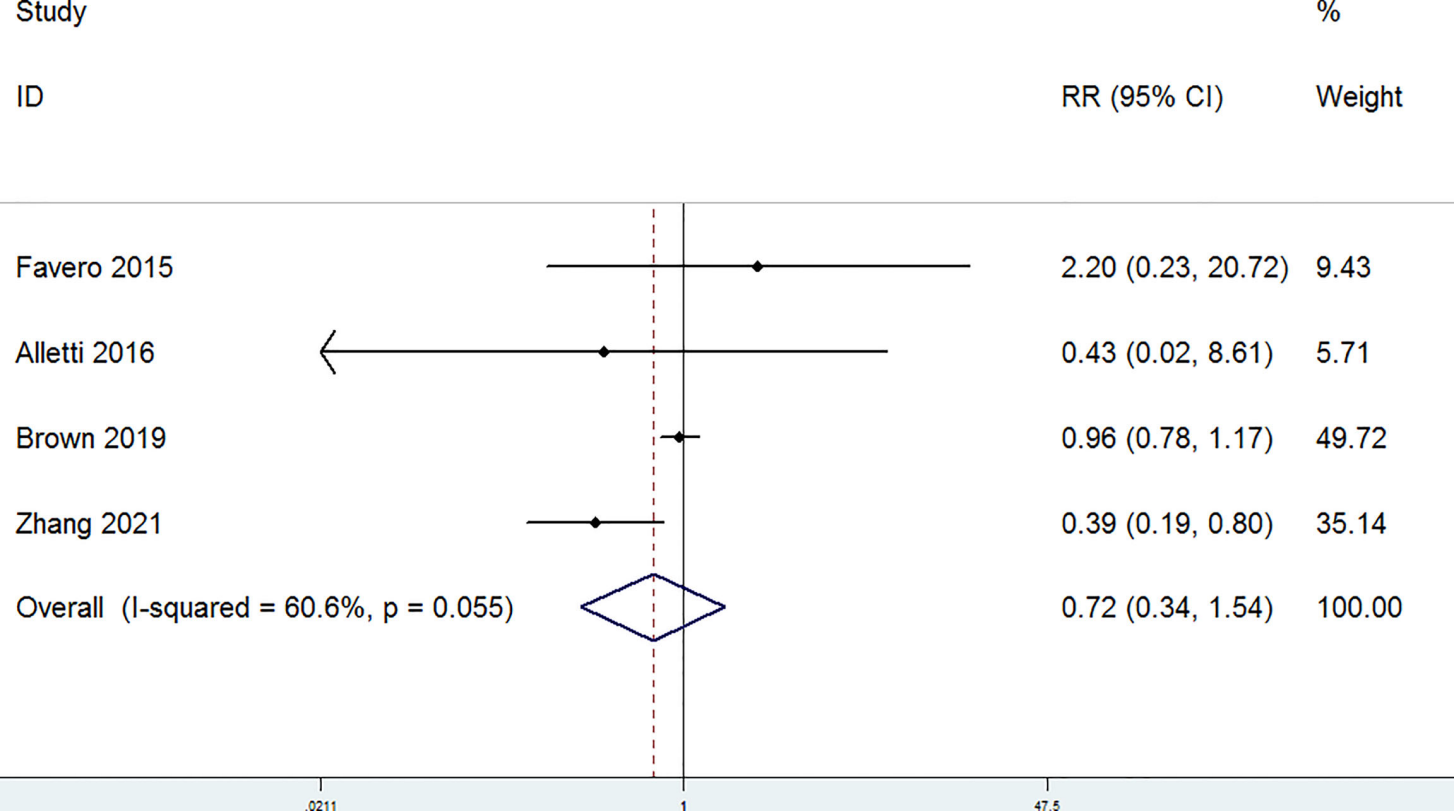
Forest plots for postoperative complications.

**Figure 7:**
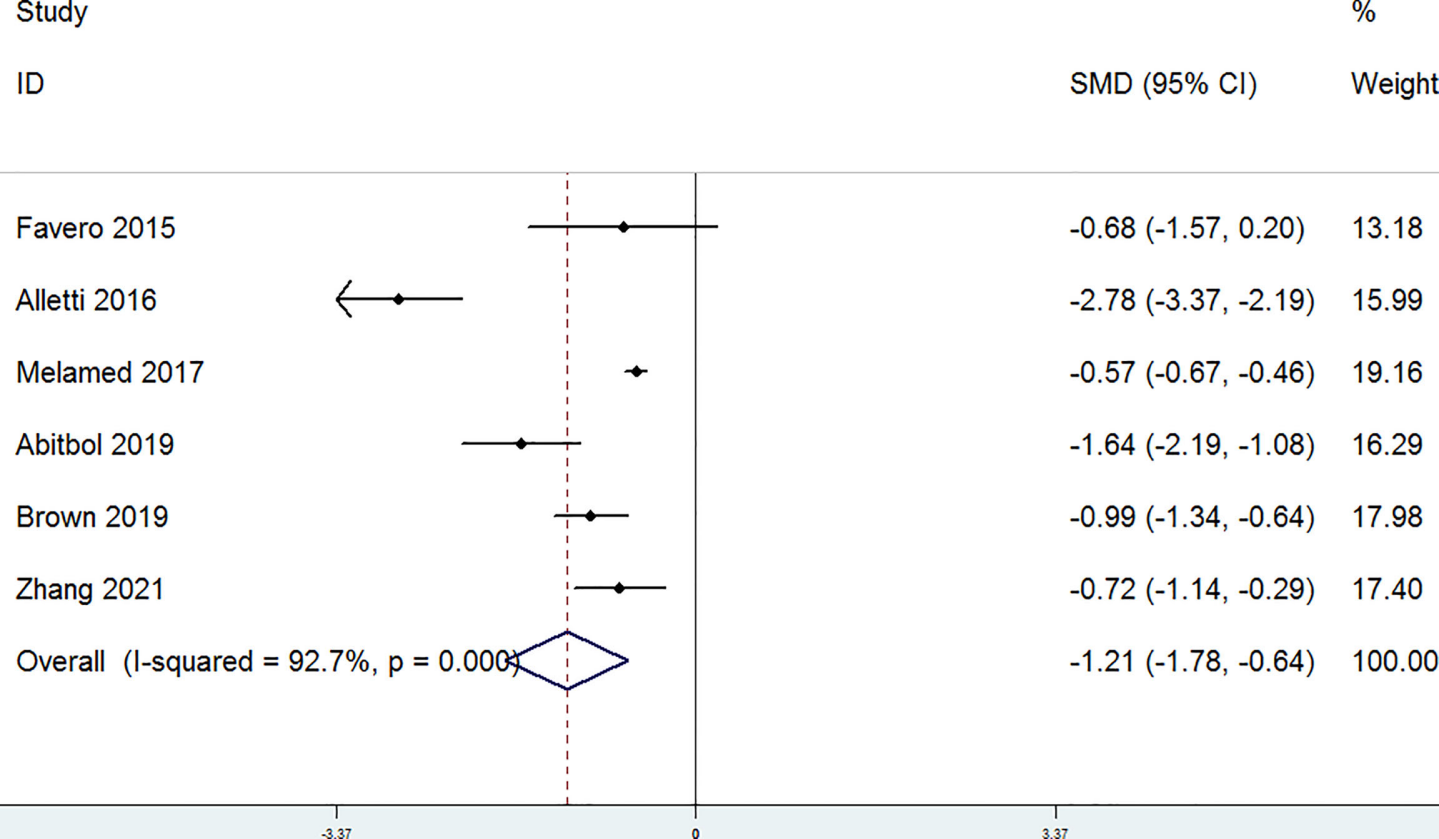
Forest plots for the length of stays in hospital.

The completeness of debulking removal (R0) of patients with OC treated with MIS versus open laparotomy was examined in four studies. The random-effect model was selected for the significant heterogeneity (*P* = 0.098, I^2^ = 52.3%). There were no significant differences in the R0 rate between patients with OC in the laparotomy group and the MIS group (RR = 1.07, 95% CI = 0.93 to 1.23) (**Figure 4**). Residual disease ≤ 1 cm (R1) was evaluated in four studies. A fixed-effect model was adopted for the non-significant heterogeneity (*P* = 0.33, I^2^ = 12.6%). According to the combined data (RR = 1.08, 95% CI = 0.91 to 1.28), R1 was similar in the MIS group and the laparotomy group (**Figure 5**). Furthermore, postoperative complications were assessed in four studies. A random-effect model was adopted (P = 0.055, I^2^ = 60.6%), and no significant differences in the incidence of postoperative complications were detected between the MIS group and the open laparotomy group (RR = 0.72, 95% CI = 0.34 to 1.54) (**Figure 6**). All the studies reported the length of stays in hospital. The length of stays in hospital was significantly shorter in patients with OC treated with MIS than those treated with open laparotomy (SMD = −1.21, 95% CI = −1.78 to −0.64; heterogeneity: *P* < 0.001, I^2^ = 92.7%) with random-effect model (**Figure 7**).

### Publication Bias and Sensitivity Analyses

As displayed in **Supplementary Figures 1**, **2**, Begg’s funnel plot indicated that both the OS (*P* = 0.103) and PFS (*P* = 0.089) did not have publication bias. Sensitivity analysis was performed by removing one study each time and assessing the influence of the remaining pooled data on the overall results. No significant changes were observed in the remaining pooled data, which suggested that our results were robust (**Supplementary Figures 3**, **4**).

## Discussion

In the present study, six studies, with 643 patients in the MIS group and 2,885 patients in the open laparotomy group, were analyzed to assess the efficacy and safety between MIS and laparotomy in IDS after NACT in patients with advanced OC. Our findings indicated that the MIS group seemed to have a better PFS than that in the open laparotomy group in patients with advanced OC, whereas no significant differences were detected in OS, completeness of debulking removal, residual disease of ≤1 cm, and postoperative complications between the two groups. Furthermore, the length of stays in hospital was significantly shorter in patients with OC who were treated with MIS than those treated with laparotomy. To our best knowledge, this was the most recent study with the largest sample size that compared the therapeutic benefits between MIS and laparotomy in patients with OC following NACT.

Although PDS was the standard treatment strategy for advanced OC, the application of NACT and IDS has increased substantially in the United States (22). It has been validated that MIS is a safe, technically practical, and appropriate procedure, as long as the optimal cytoreduction can be accomplished, not only in the case of pelvic diseases but also in the case of upper abdominal diseases (14, 16, 23). The last guideline recommends NACT and IDS for advanced patients with OC with high risk of perioperative M/M or low likelihood of attaining optimum cytoreduction at the PDS (10). In the last decade, MIS for IDS has gradually become an appealing alternative to traditional laparotomy among elderly and infirmed patients who were assessed and selected for neoadjuvant therapy. At present, assessing the feasibility of MIS for IDS after NACT have been reported in several studies (12–14, 24), but the results still remain controversial. Joel et al., for the first time, analyzed the therapeutic value of MIS and open laparotomy for IDS in patients with OC after NACT through the meta-analysis, in which the results showed that MIS appears to be a feasible and safe procedure for complete cytoreductive surgery in selected advanced patients with OC with NACT (25).

In the current study, it was also found that MIS had comparable feasibility with laparotomy for debulking OC after NACT with regarding to the OS, completeness of debulking removal, residual disease lesions, and postoperative complications, which was consistent with the previous study (11–16). At the Society of Gynecologic Oncology (SGO) conference in 2021, a retrospective study compared the surgical and tumor outcomes of MIS and open surgery in patients with advanced EOC who underwent IDS after NACT, and the results showed that MIS was a feasible and effective procedure of IDS after NACT in patients with advanced EOC (26). The proportion of reaching R0 (66% vs. 46%, P < 0.001) and optimal tumor cell reduction (93% vs. 84%, P = 0.02) was higher in MIS group. The 24-month PFS was higher in the MIS group (40% vs. 30%, P = 0.06). In addition, the ongoing prospective LANCE study would include 549 patients (27). The main purpose was to compare the PFS of MIS in IDS surgery with that in laparotomy.

Unexpectedly, our study revealed that MIS seemed to be linked with a better survival than laparotomy in patients with OC after NACT from the pooled HR for PFS. However, the sensitivity analysis showed that the PFS superiority effect was mitigated and even disappeared after removing the study of Abitbol et al. (14). In addition, almost all HRs and 95% CIs in the included study were extracted from the survival curve, which did not account for the confounding mediators between the two groups. The differences in the study design, the duration of median follow-up, and the differences in surgical techniques among gynecological oncologists do not allow to draw definitive conclusions regarding survival. For example, Alletti et al. showed that the Time to Chemo (TTC) and bevacizumab administration played independent prognostic role for PFS based on multivariable analysis, and thus, the PFS superiority effect should not erroneously ascribed to the MIS procedure only (16). On the other hand, the shorter TTC related to MIS might suggest a potential prognostic benefit of the procedure, because it ensured that the dose intensity of NACT regimens is properly maintained. In our study, there were no significant differences in NACT response between the MIS group and the laparotomy group in the original studies. Patients’ characteristics were similar among the studies. The number of neoadjuvant chemotherapy ranged from three to six cycles, and three studies reported at least a partial response before cytoreductive surgery. Because of the insufficient report on chemical cycles, ASA score, RECIST response, and other data, no detailed statistical analysis was conducted on these data. In fact, the purpose of this study was not to compare but to explore the security and effectiveness of IDS through MIS in advanced OC after NACT. Our meta-analysis suggested that MIS for IDS is feasible and safe for selecting advanced patients with OC (for partial response or complete response after NACT, optimal cytoreduction can be accomplished). This is consistent with the study on an important international multicenter experience published by Fagotti et al. They found that, for patients with AOC after NACT, MIS can be considered when the operation is limited to low complexity standard cytoreductive surgery. Meanwhile, MIS is associated with R0 recovery rate of 96%, and the median PFS and OS reported in this series seem reassuring (28). The SGO meeting in 2021 also reported the effects of laparoscopic prediction of minimally invasive IDS for advanced OC (MIID-SOC Test) with a clinical model, which calculated the total predictive index score (PIV) according to the location of pelvic and peritoneal lesions. The study showed that, when the PIV is less than 2, patients may achieve the best effects of minimally invasive IDS (29). However, whether this clinical prediction model can be used in clinical practice still needs to be verified by randomized controlled trials. Therefore, in this context, the finding of a better PFS in patients treated with MIS needs to be further clarified with large-scale randomized clinical trial with respect to standard laparotomy.

The secondary outcomes of the study proved that the length of stays in hospital was significantly shorter in patients with OC who were treated with MIS than those treated with laparotomy. In addition, although MIS did not reach a statistical significance, it had a trend to reduce postoperative complications, including bleeding, fever, infection, cardiac complications, ileus, lymphocyst formation, urinary retention, and wound complications (11–13, 30). This phenomenon might be explained as the following reasons: (1) small sample size; (2) heterogeneous literature; and (3) hynecologists have different surgical techniques.

## Limitations

Some limitations in our meta-analysis should be noted. First, the limited included studies had relatively small sample sizes. Second, the heterogeneity of study design, surgical procedure, and chemotherapy regimen in the included studies might result in potential biases. Third, only English-language published literature studies were included, which may lead to missing data. Fourth, there are insufficient data to evaluate the chemotherapy response, chemical cycles, and ASA score. Fifth, our literature only confirmed the results of current literature studies and provided another option for traditional IDS. This study did not add specific value to the scientific knowledge about the role of MIS in IDS.

## Data Availability Statement

The datasets presented in this study can be found in online repositories. The names of the repository/repositories and accession number(s) can be found below: PubMed, Embase, and the Cochrane Library.

## Author Contributions

SZ, Conceptualization, Methodology, Data curation, Writing- Original draft preparation, Visualization, Software, investigation, writing - review and editing. YY, YC, BL and ZL conceptualization, investigation, ,writing-review and editing. LL and XJ, final approval of the version, writing- review and editing.

## Conflict of Interest

The authors declare that the research was conducted in the absence of any commercial or financial relationships that could be construed as a potential conflict of interest.

## Publisher’s Note

All claims expressed in this article are solely those of the authors and do not necessarily represent those of their affiliated organizations, or those of the publisher, the editors and the reviewers. Any product that may be evaluated in this article, or claim that may be made by its manufacturer, is not guaranteed or endorsed by the publisher.

## Supplementary Material

Supplementary Figure 1 Publication bias detected by Begg’s funnel plots for OS.

Supplementary Figure 2 Publication bias detected by Begg’s funnel plots for PFS.

Supplementary Figure 3 Sensitivity analysis for the meta-analysis (OS).

Supplementary Figure 4 Sensitivity analysis for the meta-analysis (PFS).
