# Supplementary figures and images for: Efficacy and Safety of Minimally Invasive Surgery Versus Open Laparotomy for Interval Debulking Surgery of Advanced Ovarian Cancer After Neoadjuvant Chemotherapy: A Systematic Review and A Meta-Analysis
Source: Front Oncol. 2022 Jul 18;12:900256. doi: 10.3389/fonc.2022.900256 (PMC9341245; doi:10.3389/fonc.2022.900256)

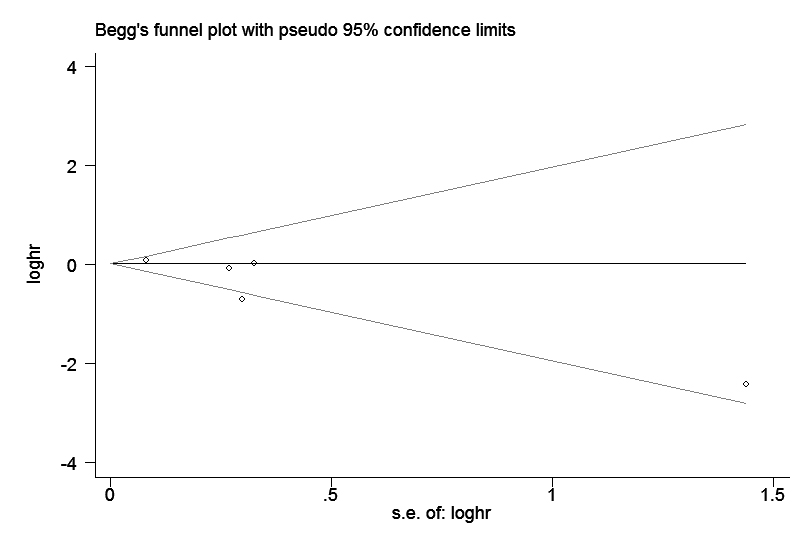

Supplement: Supplementary Figure 1 — Publication bias detected by Begg’s funnel plots for OS. [file Image_1.jpeg]

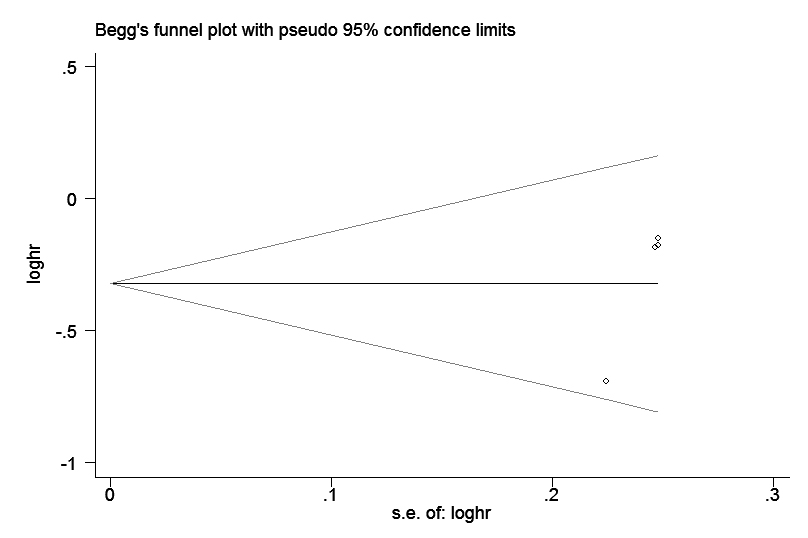

Supplement: Supplementary Figure 2 — Publication bias detected by Begg’s funnel plots for PFS. [file Image_2.jpeg]

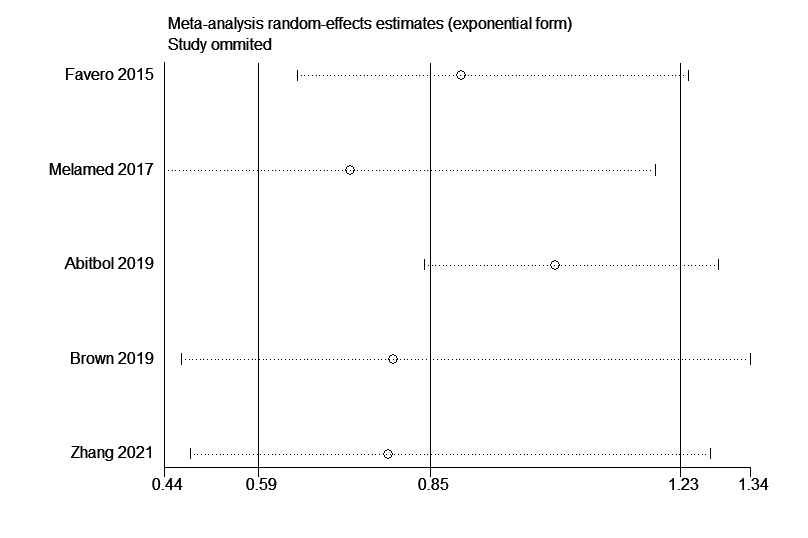

Supplement: Supplementary Figure 3 — Sensitivity analysis for the meta-analysis (OS). [file Image_3.jpeg]

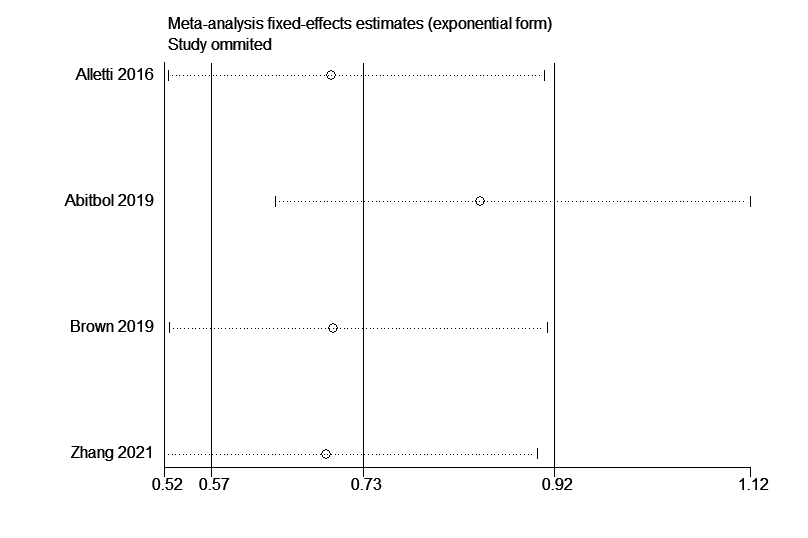

Supplement: Supplementary Figure 4 — Sensitivity analysis for the meta-analysis (PFS). [file Image_4.jpeg]
